# Best Practices in Structural Ensemble Analysis: Avoiding Pitfalls, Interpreting Results, and Automating Workflows with EnsembleFlex

**DOI:** 10.1002/cpz1.70345

**Published:** 2026-03-20

**Authors:** Melanie Schneider, José Antonio Marquez, Andrew R. Leach

**Affiliations:** ^1^ European Molecular Biology Laboratory EMBL‐EBI, Wellcome Genome Campus Hinxton United Kingdom; ^2^ European Molecular Biology Laboratory EMBL Grenoble France

**Keywords:** automated workflows, PCA, protein flexibility, rigid‐core superposition, structural ensemble analysis

## Abstract

Structural ensemble analysis is critical for understanding protein flexibility, function, and interactions. EnsembleFlex provides a streamlined framework for dual‐scale (backbone‐only and all‐atom) flexibility analysis by integrating both data‐driven and predictive approaches. This article outlines best practices for analyzing protein ensembles, with detailed guidance on avoiding common pitfalls, interpreting outputs from both backbone‐only and all‐atom assessments (including RMSD, RMSF, PCA, and UMAP plots), and automating workflows for reproducibility. The framework is designed to require minimal user adjustments, allowing researchers to adapt the protocol to various datasets and research objectives with ease. © 2026 The Author(s). *Current Protocols* published by Wiley Periodicals LLC.

**Basic Protocol**: Best practices for structural ensemble analysis

## INTRODUCTION

Protein flexibility underpins essential biological functions such as molecular recognition, enzyme catalysis, and signal transduction. Traditional methods capture static structures, yet dynamic ensemble analysis reveals how conformational variability influence's function. However, analyzing heterogeneous ensembles, with variable atom counts, missing residues, and sequence differences, remains challenging. Many current methods focus solely on either computationally generated [e.g., molecular dynamics (MD) simulations or normal mode analysis (NMA)‐based models] or experimentally derived ensembles, rarely providing an integrated approach to dissect both global and local flexibility. EnsembleFlex addresses this gap with a dual‐scale analysis: backbone‐only assessments capture large‐scale domain motions, while all‐atom analyses reveal fine side‐chain dynamics (Schneider et al., 2025). Moreover, EnsembleFlex incorporates advanced superposition strategies (including a robust rigid‐core method) and a maximally automated workflow that requires only minimal user adjustments, making it highly adaptable to diverse datasets and research aims. This article offers detailed recommendations to avoid pitfalls, interpret output plots, and effectively harness EnsembleFlex for structural ensemble analysis.

## STRATEGIC PLANNING

Although EnsembleFlex is designed for rapid and automated analysis, careful planning can enhance reproducibility and result interpretation. Users should ensure that input and output folders are clearly defined to prevent file overwriting. For multimeric structures, chain splitting and subsetting are essential steps. A preliminary alignment overview can help determine if additional preprocessing is needed based on the dataset's heterogeneity (e.g., filtering of incomplete structures).

## BEST PRACTICES FOR STRUCTURAL ENSEMBLE ANALYSIS USING EnsembleFlex

This protocol outlines a recommended approach for analyzing protein structural ensembles with EnsembleFlex. It emphasizes comprehensive data preparation, advanced superposition strategies, and double‐scale flexibility analysis. Detailed steps guide the user through avoiding common pitfalls (e.g., misalignment in heterogeneous ensembles) and interpreting results using multiple metrics, such as RMSD, RMSF, PCA, and UMAP. The automated nature of EnsembleFlex ensures minimal adjustments are necessary, allowing researchers to focus on data interpretation.

### Necessary Resources

#### Hardware

Standard desktop or laptop with Linux, macOS, or Windows (with Docker/Conda support)

##### Software

EnsembleFlex (available on GitHub)
Python and R (dependencies as per environment.yml and requirements.R).

##### Files

Input folder with monomeric PDB files; sample datasets available from the EnsembleFlex repository

*NOTE*: The protocol steps can be performed through the EnsembleFlex interface, but the user has also the option to use the provided scripts through the command line to set up a fully automated custom workflow. The provided example commands in this protocol generally follow the same format: <python3/Rscript> <path_to_script> ‐i <input_folder> ‐o <output_folder> [<additional_flags/options>].

#### Condensed High‐Level Workflow Steps

1 Clone repository and Install environment (Conda recommended):


git clone https://github.com/chembl/ensembleflex.git ~/ensembleflex
conda env create -f environment.yml -n ensembleflex_env
conda activate ensembleflex_env
export ENSEMBLEFLEX_HOME=~/ensembleflex

2 Prepare input. Collect monomeric PDB files into a directory <input_dir> (to split multimers use Rscript $ENSEMBLEFLEX_HOME/src/tools/split_pdbs_bio3d.R ‐i <multimer_pdb_dir> ‐o <input_dir>).
3 Rigid‐core superposition:


Rscript $ENSEMBLEFLEX_HOME/src/superimpose_bio3d.R ‐i <input_dir> ‐o <superimp_out_dir>

4 Main flexibility analysis:


Rscript $ENSEMBLEFLEX_HOME/src/analyse_flex_bio3d.R ‐i <superimp_out_dir> ‐o <analysis_dir> ‐n <n_clusters>

5 Optional modules: SASA difference analysis, ligand‐site analysis, conserved‐water analysis, and ENM predictions are available as separate scripts (see below in “Full explanation of workflow steps” and the README for full options).

#### Full Explanation of Workflow Steps

##### 1. Installation and environment setup

EnsembleFlex is distributed through a public GitHub repository and can be installed locally using either Conda or Docker. All helper scripts, workflows, and example datasets are included in the repository.

1 Obtain the source code:


git clone https://github.com/chembl/ensembleflex.git ~/ensembleflex
export ENSEMBLEFLEX_HOME=~/ensembleflex

  All scripts referenced in this protocol are in the src/ and src/tools/ directories of the cloned repository.
2a Conda‐based installation (recommended). A Conda environment specification file (environment.yml) is provided for straightforward installation:


conda env create ‐f environment.yml ‐n ensembleflex_env
conda activate ensembleflex_env

  For fully version‐pinned reproducibility, a second environment file (environment_versioned.yml) is provided. Users wishing to recreate the exact tested software environment should install using:


conda env create ‐f environment_versioned.yml ‐n ensembleflex_env
conda activate ensembleflex_env

  For Linux and macOS systems, a conda‐lock.yml file is included. This lock file ensures byte‐for‐byte reproducibility of the environment across installations:


conda‐lock install --name ensembleflex_env conda‐lock.yml
conda activate ensembleflex_env
2b Docker‐based installation. A Dockerfile is included in the repository to allow containerized execution. Because EnsembleFlex is not distributed through DockerHub, users must build the image locally:


docker build ‐t ensembleflex

  The container can then be launched with:


docker run ‐d ‐it --name ensembleflex ‐p 8501:8501 ‐p 80:80 --volume ./docker‐data:/app/docker‐data ensembleflex‐image

  All EnsembleFlex scripts and example datasets are available inside the container at:


/docker‐data
  To access the graphical user interface, open your browser and navigate to: http://localhost:8501/.

###### 2. Data preparation and preprocessing

1 Input data selection: Ensure your dataset comprises monomeric PDB files. For multimeric proteins, use the provided split_pdbs_bio3d.R script to separate chains.
  Example:


> Rscript $ENSEMBLEFLEX_HOME/src/tools/split_pdbs_bio3d.R ‐i multimer_pdbs ‐o pdbs

  This step is critical for heterogeneous multimeric ensembles where differences in chain composition and missing residues can hinder accurate analysis.
2 Filtering: Optionally remove structures with significant missing residues using the filtering scripts provided.
  Example:


python3 $ENSEMBLEFLEX_HOME/src/tools/sort_pdbs_has_gap_in_pdb_seq.py ‐i pdbs/ ‐o
3 Consolidate the chains of interest into a dedicated folder for analysis.

###### 3. Superposition

Rigid‐core superposition: Execute superimpose_bio3d.R to perform rigid‐core superposition, provided through Bio3D (Grant et al., 2021).

Example:


> Rscript $ENSEMBLEFLEX_HOME/src/superimpose_bio3d.R ‐i pdbs ‐o EnsembleFlex



This method iteratively refines the alignment from an initial multiple sequence alignment by excluding positions with the highest spatial deviations. Variability is quantified via eigenvalues of Cartesian coordinates; the variance ellipsoid's volume serves as a measure of local structural variation, thereby identifying the most invariant region, i.e., the rigid core. This focused approach enhances downstream flexibility assessments by reducing noise from variable regions. An alternative iterative alignment via ProDy (Bakan et al., 2011; Zhang et al., 2021) is also available, where the ensemble is repeatedly aligned to a recalculated mean reference until convergence (as measured by RMSD changes).

Note that the subsequent analysis results may differ substantially depending on the superposition method used. We generally advise using Bio3D's rigid core method. It is most suited for subsequent detection of variable areas, as it disregards the variable areas for superpositioning.

Comparison with other alternative methods: In contrast to EnsembleFlex's targeted approach, PDBe's GESAMT (Krissinel & Uski, 2017) maximizes residue matches under an RMSD threshold for rapid global alignments, and RCSB‐PDB's FATCAT (Li et al., 2020) segments proteins to accommodate conformational variability. While robust for database searches, these methods may obscure subtle local dynamics. For ligand‐bound ensembles, consider binding site‐focused superposition to preserve local alignment of residues surrounding the binding pocket.

###### 4. Flexibility analysis

1 Double‐scale flexibility analysis: Run analyse_flex_bio3d.R to perform the main flexibility analysis, supplying the desired number of clusters (with flag “‐n”).
  Example:


> Rscript $ENSEMBLEFLEX_HOME/src/analyse_flex_bio3d.R ‐i EnsembleFlex/superimposed ‐o EnsembleFlex/Analysis_Bio3D ‐n 3

  The script performs several types of calculations in a streamlined and coherent way. (A) RMSD and RMSF are calculated. Backbone‐only analysis captures global motions (e.g., domain shifts, flexible loops), while all‐atom analysis reveals fine side‐chain fluctuations that are critical for ligand interactions. Using both analyses together provides complementary insights, i.e., backbone assessments clarify overall structural shifts, and all‐atom assessments highlight local, functionally relevant variations. (B) Dimensionality reduction is performed by applying PCA and UMAP to decompose structural variability into dominant motion modes. PCA not only visualizes the major conformational changes (including per‐residue contributions and the explained proportion of variance per PC) but also enables 3D animations along principal component vectors. UMAP, with its nonlinear capabilities, preserves complex relationships in high‐dimensional data and is therefore complementary to PCA. The combination of these methods, followed by clustering, helps identify distinct conformational states. Clustering is automatically performed on both backbone and all‐atom metrics, with results summarized in a comparative plot. This allows the user to assess agreements or discrepancies between different granularity levels.
2 Subset structures by clustering: To subset structures according to one of the clustering results, use the provided sorter module pdb_sorter_from_dataframe.py with supplying with flag “‐d” the clustering result datafile and with flag “‐c” the column name/clustering method.
  Example:


> python3 $ENSEMBLEFLEX_HOME/src/tools/pdb_sorter_from_dataframe.py ‐i EnsembleFlex/superimposed ‐o EnsembleFlex/Analysis_Bio3D/Consensus_Clusters ‐d EnsembleFlex/Analysis_Bio3D/cluster_attributions_with_consensus.csv ‐c backbone_PCA_onCoords

###### 5. Environment change analysis (SASA difference analysis)

1 Execute analyse_flex_sasa_biopython.py to compute the solvent accessible surface area (SASA) for each residue across the ensemble. Algorithm provided by Biopython (Cock et al., 2009).
  Differences and standard deviations in SASA values yield insights into local environmental changes, such as those resulting from conformational shifts or ligand‐induced rearrangements.
2 Generate an additional visual output of annotated PDB files with data_on_structure.R.
  This complements flexibility metrics and assists in identifying regions of altered solvent exposure.

###### 6. Ligand binding site analysis

1 Preprocessing: In the PDB data file format each atom is designated either ATOM or HETATM (which stands for hetero atom). ATOM is used for atoms in standard residues and HETATM is used for non‐standard residues, and atoms in other kinds of groups, such as carbohydrates, substrates, ligands, solvent, and ions.
  A user may want to remove unwanted HETATM molecules (ions, crystallization additives) using run_pdb_del_on_directory.sh with scripts like pdb_delhetatm_ions.py and pdb_delresname.py before subsetting structures containing ligands using pdb_sorter_has_ligand.R.
2 Identification and analysis: Run identify_binding_site_bio3d.R to automatically detect ligand binding sites based on a distance cutoff (default 3.5 Å) and generate ligand‐residue interaction heatmaps. Subsequently, execute analyse_flex_binding_site_bio3d.R to calculate RMSF for binding site residues; the results are annotated in the B‐factor column to facilitate visualization.
  This dual approach reveals both the structural dynamics of binding sites and their potential impact on ligand‐binding stability.

###### 7. Conserved water analysis

1 Run analyse_conserved_waters.R to identify water molecules that are consistently present across the ensemble. The vanddraabe package (vanddraabe: Statistical Analysis of Conserved Waters) clusters water molecules based on spatial proximity (grouping oxygen atoms within 2.4 Å) and correlates their conservation with ligand binding patterns.
  Two PyMOL scripts are automatically generated to visualize these conserved waters: (1) globally to provide an overview of water conservation across the whole protein and detect highly conserved clusters; and (2) in the context of a ligand binding site, thereby highlighting potential water‐mediated interactions that may be critical for rational drug design.

###### 8. Flexibility prediction (optional)

These predictive approaches complement experimental data, providing insights into intrinsic motion patterns and potential allosteric sites.

1 Coarse‐grained NMA: Execute predict_flex_nma_bio3d.R to perform normal mode analysis (NMA) on Cα atoms, providing a global perspective on protein dynamics.
2 All‐atom NMA: Run predict_flex_aanma_bio3d.R to capture fine‐scale, all‐atom motions, noting that this analysis is computationally intensive and recommended only for small ensembles.
3 ESSA: Use predict_flex_binding_site_essa_prody.py to conduct essential site scanning analysis (ESSA), which identifies key residues whose perturbation would significantly alter the protein's overall dynamics.

#### Tested environments and reproducibility

The EnsembleFlex workflows described in this protocol were developed and tested on Linux and macOS using Python 3.11 and R 4.3.3. Principal dependencies include Bio3D (R), ProDy (Python), and Biopython. To ensure reproducibility, the repository includes:

environment.yml (general Conda environment specification)
environment_versioned.yml (fully version‐pinned Conda environment)
conda‐lock.yml (lock file for exact package resolution on Linux/macOS).

Users are strongly encouraged to install EnsembleFlex using environment_versioned.yml or conda‐lock.yml to reproduce the tested computational environment precisely. The repository includes a Dockerfile as an alternative containerized option to promote reproducible deployments across platforms.

#### Sample files

Two sample datasets are provided in the docker‐data/examples folder of the EnsembleFlex repository.

## COMMENTARY

### Background Information

This protocol consolidates best practices in structural ensemble analysis using EnsembleFlex. By combining backbone‐only and all‐atom analyses, the method addresses the limitations of traditional approaches. Advanced superposition techniques (e.g., rigid‐core alignment) and automated scripting significantly improve reproducibility and efficiency, providing comprehensive insights into both global and local dynamics.

### Critical Parameters

Key factors include the quality of input PDB files, selection of the superposition method, the clustering threshold, and distance cutoffs for binding site detection. These parameters directly influence the sensitivity and accuracy of the analysis.

### Troubleshooting

Table 1 lists common issues, possible causes, and solutions.

**Table 1:** Sources and Solutions to Potential Errors

Problem	Possible Cause	Solution
The residues of interest are omitted/not shown in the analysis	The residues of interest may not be resolved/present in all structures	Create a data subset containing only the structures with fully resolved residues of interest and analyze them separately
One of the first steps (alignment or analysis) fails	There may be structures in your dataset that cannot be read properly	Create data subsets and run the pipeline on those to find out which structures cause the problem and either exclude them from analysis or try to investigate and fix the cause in the file(s)
The alignment with Bio3D aligns the structures on one domain, but another domain is desired	The atoms used for the alignment with the Bio3D rigid core algorithm are usually located in the largest domain; the selection is automatic	Skip the automatic alignment procedure and provide a custom pre‐aligned ensemble for analysis or use the dedicated binding‐site alignment tool that allows for supplying specific residues for alignment

### Understanding Results

This section provides detailed instructions for interpreting output plots generated by EnsembleFlex. For example, RMSD and RMSF plots reveal global and local flexibility, respectively.

#### RMSD and RMSF

RMSD measures the overall structural deviation between two conformations of a protein. It quantifies the average distance between the atoms of a target structure and a reference structure after optimal superimposition. A higher RMSD value indicates a greater structural difference between the two conformations. RMSD is, however, sensitive to large displacements of small regions (e.g., mobile loops), which can dominate the metric and obscure more subtle, distributed motions.

RMSF measures the fluctuation of individual atoms or residues around their average positions within an ensemble or trajectory. It provides a residue‐level measure of mobility and highlights the most labile regions of the protein. RMSF values are straightforward to visualize as per‐residue color maps (e.g., by writing values into the B‐factor column) to localize dynamic hotspots.

RMSD and RMSF are complementary: RMSD captures global structural changes, while RMSF highlights local flexibility. Interpretation depends on the superposition strategy, reference choice, and ensemble size; always report these choices alongside the metrics.

#### Analysis on two scales: Backbone‐only vs all‐atom flexibility

EnsembleFlex computes flexibility at two scales and interpreting them together is essential for correct biological interpretation.

Backbone‐only (N, Cα, C, O), or even Cα‐only, captures large‐scale, collective motions of the protein scaffold, i.e., domain translations, hinge bending, and loop movements. Backbone metrics are directly comparable between residues and across ensembles because the same atoms are present for each residue. This scale is therefore ideal for identifying domain motions and hinge axes.

All‐atom (includes side‐chain heavy atoms) provides detailed residue‐level information, revealing rotameric changes, side‐chain breathing of pockets, and local packing rearrangements that are crucial for ligand recognition and catalysis. All‐atom metrics are influenced by residue size, atom count, and local environment, and can therefore show elevated values for bulky or solvent‐exposed residues even in the absence of backbone displacement.

Interpretation rule‐of‐thumb: Always consider all‐atom results in the context of backbone results. If backbone RMSF is high for a residue, high all‐atom RMSF is expected and likely reflects true backbone mobility. If backbone RMSF is low but all‐atom RMSF is high, the mobility is predominately side‐chain‐driven and may affect ligand interactions without large backbone rearrangements. Using both scales together enables discrimination between global architectural changes and local, functionally relevant rearrangements.

#### Difference‐based descriptors (torsion angles, distance matrices, eDDM)

EnsembleFlex implements difference‐based metrics that are independent of superposition. Torsion‐angle differences (backbone φ/ψ) are often more sensitive to local conformational changes than Cartesian coordinates and are robust to rigid‐body displacements. All‐atom difference distance matrices capture side‐chain rearrangements and local packing changes. The ensemble difference distance matrix (eDDM) analysis in EnsembleFlex provides a dedicated, pairwise statistical test to identify significant differences between structural subgroups. Use these superposition‐independent checks to validate coordinate‐based observations.

#### Dimension reduction: Rationale and use

High‐dimensional descriptors (atomic coordinates, RMSD matrices, torsion‐angle vectors) are difficult to visualize directly. Dimension reduction provides low‐dimensional embeddings that preserve key relationships and enable intuitive 2D/3D plots for exploratory analysis.

EnsembleFlex supports:
  (1) Coordinates (backbone or all‐atom). Coordinate‐based PCA retains information about collective Cartesian motions and is well suited to detect domain movements and hinge‐like behavior. Input structures must be consistently aligned before PCA.
  (2) RMSD/distance matrices. Dimension reduction on pairwise distance/RMSD matrices emphasizes relative conformational dissimilarities and is relatively robust to alignment issues when difference‐distance descriptors are used.
  (3) Torsion‐angle vectors (backbone φ/ψ). Torsion‐space embeddings are sensitive to local backbone rearrangements and rotameric differences; they can reveal substates not evident in Cartesian PCA.

#### Principal component analysis (PCA)

PCA is a linear technique identifying orthogonal modes (principal components, PCs) that capture decreasing proportions of variance. In EnsembleFlex, PCA applied to aligned backbone coordinates is commonly used to extract dominant collective motions.

Key practical points:
  (1) Explained variance. Inspect the percentage of variance explained by the first PCs. If PC1 and PC2 capture a large fraction (e.g., >50% to 70%), a few modes summarize most variation; if explained variance is low, more PCs are needed to characterize the ensemble.
  (2) PC scatter plots. Projecting structures onto PC axes (e.g., PC1 vs PC2) highlights clusters and putative transition trajectories.
  (3) Per‐residue loadings. Decompose PCs to determine which residues contribute most. Mapping loadings onto the structure reveals hinge regions and moving domains.
  (4) Animations and extrapolation. Animate motions along PCs to visualize directions of motion. Be aware that those extrapolations may produce non‐physical conformations.
  (5) Pitfalls: PCA on poorly superposed structures produces spurious modes. PCA results can also be biased by ensemble composition (over‐representation of one state).

#### UMAP and non‐linear embeddings

UMAP is a non‐linear embedding preserving local neighborhoods and revealing complex multi‐modal structure. Use UMAP when the conformational landscape is expected to be non‐linear or multi‐peaked (e.g., multiple bound substates). Note UMAP's sensitivity to hyperparameters (n_neighbors, min_dist) and random initialization. Generally, we advise prioritizing PCA instead of UMAP for dimension reduction.

#### Clustering: Purpose and recommended practice

Clustering groups conformations into structurally similar states, facilitating downstream interpretation and decision‐making (e.g., selecting representative conformations for docking).

EnsembleFlex performs clustering on multiple descriptors (backbone RMSD, PCA coordinates, UMAP embeddings) and generates automated cluster‐number estimation reports (connectivity, Dunn index, Silhouette) using clValid.

Recommendations:
  (1) Choose descriptor to match the question. Backbone PCA for domain‐level motions; torsion‐space clustering for local active‐site substates.
  (2) Assessing cluster number and stability. EnsembleFlex provides automated cluster‐number estimation (connectivity, Dunn index, Silhouette) via clValid across hierarchical, k‐means, and PAM algorithms. Use these reports as guidance but also manually inspect cluster validity. Compare clustering across descriptors and algorithms to evaluate robustness.
  (3) Inspect representatives. For each cluster, examine centroid/medoid structures to characterize structural differences and to select representative models for follow‐up.
  (4) Beware small clusters. Small clusters may represent noise or rare, biologically relevant substates. Try to validate those using other metrics before discarding.

#### Common pitfalls and caveats

(1) Superposition dependency. Coordinate PCA depends critically on the quality of alignment; poor superposition (particularly in heterogeneous ensembles) can yield artifactual PCs.
(2) Over‐interpretation of low‐variance PCs. PCs with very low explained variance (<1% to 2%) may still be meaningful but require caution, as they may also just represent noise.
(3) Sampling bias and ensemble size. Small or biased ensembles (e.g., dominated by one crystal form) limit interpretability; strive for balanced representation of states where possible.
(4) Descriptor mismatch. Different descriptors emphasize different aspects of motion; a combined, comparative approach is recommended.

#### Interpreting combined analyses

Together the provided metrics enable a landscape overview of the variability present in an ensemble. Users should compare the provided metrics and evaluate the significance of a metric in relation to the others for a given ensemble. For robust conclusions, synthesize results across methods: overlay RMSF hotspots with residues that have high PC loadings; check whether clusters identified by PCA correspond to discrete torsion‐angle substates; and validate coordinate‐based clusters using eDDM and torsion differences as superposition‐independent evidence.

#### Ligand binding site analysis

EnsembleFlex's automated binding‐site module identifies residues within a user‐defined heavy‐atom distance cutoff (default 3.5 Å) from HETATM ligand atoms and summarizes occurrence frequencies, ligand‐residue heatmaps, and annotated PDBs (B‐factor encoding of occurrence/frequency). When interpreting these outputs, combine frequency information with flexibility metrics: residues that are frequently observed in the binding site and show low backbone and all‐atom RMSF are likely structurally stable anchors for ligand interactions; and residues with high binding frequency but high RMSF indicate flexible contacts that may confer promiscuity or reduce affinity. Use the ligand‐residue heatmap and clustered interaction patterns to detect sub‐pocket preferences and to prioritize fragment series that share conserved interaction fingerprints. Check binding‐site radius of gyration (an approximation to binding‐site‐size) histograms to assess site plasticity: a narrow distribution suggests a rigid/fixed site, while a broad distribution signals adaptable binding sites that may require different optimization strategies.

#### Conserved‐water analysis

Conserved‐water analysis (clustered oxygen positions, typically within 2.4 Å) highlights hydration sites that mediate protein‐ligand contacts or stabilize pocket geometry. Visualize conserved water clusters with the provided PyMOL scripts. Waters conserved across many structures are good candidates for exploitation or displacement in fragment growth, whereas partially conserved waters may be context‐dependent and useful for ligand design that preserves favorable water‐mediated networks. Always inspect crystallographic metadata (resolution, occupancy, alternate conformations) and filter spurious HETATMs/ions before analysis since crystallization additives can produce misleading binding‐site or water clusters. Finally, cross‐validate binding‐site signals with ESSA/ENM outputs: residues identified as ESSA hotspots that also appear in frequent binding‐site mappings point to positions where ligand binding could materially alter global dynamics and are high‐priority targets for experimental follow‐up.

#### Predictive (ENM‐based) methods: ANM, GNM and ESSA

In addition to data‐driven ensemble analyses, EnsembleFlex integrates predictive, generative methods based on elastic network models (ENMs) to provide complementary insight into intrinsic protein dynamics. The tool implements two commonly used coarse‐grained ENMs, i.e., Gaussian network model (GNM) and anisotropic network model (ANM), as well as all‐atom normal mode analysis (aaNMA) and essential site scanning analysis (ESSA).

##### GNM (Gaussian network model)

GNM provides estimates of the magnitude of residue fluctuations (scalar fluctuation profile) and residue‐residue cross‐correlation patterns based on a coarse‐grained Cα network. GNM is computationally efficient and useful for predicting relative flexibility and correlated regions, but it does not provide directionality of motion.

##### ANM (anisotropic network model)

ANM extends ENM analysis by yielding vectorial normal modes that describe both amplitudes and directions of collective motions. ANM is therefore better suited for investigating the mechanisms and directions of domain movements (e.g., hinge bending, domain opening) while remaining computationally inexpensive compared to atomistic simulations.

##### All‐atom NMA (aaNMA)

When finer detail is required, EnsembleFlex can run all‐atom normal mode analyses (via Bio3D) to capture side‐chain and local packing contributions to low‐frequency motions. aaNMA is more computationally demanding and can be sensitive to the starting structure; interpret results with caution when ensemble diversity is high.

##### ESSA (essential site scanning analysis)

ESSA evaluates the effect of perturbing (e.g., ligand‐binding) individual residues on the global dynamics predicted by an ENM. It identifies residues that, if bound or perturbed, are predicted to significantly alter collective motions, thereby suggesting potential allosteric hotspots or sites of functional importance.

##### Interpretation and best practices

ENM‐based predictions are valuable when experimental ensembles are sparse or biased, as they highlight intrinsic tendencies of the fold to undergo collective motions. However, ENMs are reference‐structure dependent and reflect the topology encoded in the input structure; therefore, always compare ENM predictions (mode shapes, fluctuation profiles, ESSA hotspots) with data‐driven metrics (PCA modes, RMSF, eDDM). Use mode overlap measures or visual comparison of ANM mode vectors with PCA/extrapolation animations to assess concordance. Discrepancies can be informative: where ENM predicts motion not observed in the experimental ensemble, consider experimental constraints (crystal contacts, ligand stabilization) or limited sampling as possible explanations.

### Suggestions for Further Analysis

Upon analysis of an experimental ensemble dataset a user may want to consider generating a computational ensemble starting from a structure of interest, identified through the EnsembleFlex analysis, and repeat the analysis on such an ensemble, to further deepen the insights in the direction of interest.

In a fragment/ligand screening setup a user may want to consider performing additional fine‐grained protein‐ligand interaction analysis.

### Author Contributions

**Melanie Schneider**: Conceptualisation; data curation; investigation; methodology; software; validation; writing—original draft; writing—review and editing. **José Antonio Marquez**: Supervision. **Andrew Leach**: Supervision.

### Conflict of Interest

The authors declare no competing interests.

## Data Availability

The EnsembleFlex code is available on GitHub at: https://github.com/chembl/ensembleflex.git, with environment files and the Dockerfile. Detailed installation instructions and sample datasets are included in the repository. All code and data are distributed under the MIT license. EnsembleFlex has been published in the journal Structure with example analyses and images (Schneider et al., 2025).
